# Rubella virus-associated uveitis at a tertiary care hospital in Germany between 2013 and 2019

**DOI:** 10.1186/s12886-023-03182-y

**Published:** 2023-11-06

**Authors:** Mario Hönemann, Elizabeth Scharfenberg, Nadine Dietze, Claudia Claus, Claudia Jochmann, Uwe Gerd Liebert

**Affiliations:** 1 Institute of Medical Microbiology and Virology, Leipzig University, Johannisallee 30, 04103 Leipzig, Germany; 2 Department of Ophthalmology, University Hospital Leipzig, Leipzig University, Liebigstrasse 21, 04103 Leipzig, Germany

**Keywords:** Rubella virus, Uveitis, Fuchs Uveitis, Ocular Infection

## Abstract

Uveitis is a process of intraocular inflammation that may involve different sections of the uveal tract. Apart from systemic or localized immune-mediated diseases, infections are key players in the etiology of uveitis and entail different treatment strategies. Rubella virus (RuV) is a recognized causative agent for the development of Fuchs uveitis, representing a major cause of virus-associated intraocular inflammation. A cohort of 159 patients diagnosed with different forms of uveitis between 2013 and 2019 was subjected to diagnostic antibody testing of the aqueous or vitreous humor. The diagnostic panel included RuV, cytomegalovirus, herpes simplex virus, varicella-zoster virus, and toxoplasmosis. Within this cohort, 38 RuV-associated uveitis (RAU) patients were identified based on a pathologic Goldman-Witmer coefficient indicative of an underlying RuV infection. With a mean age of 45.9 years, the RAU patients were younger than the non-RAU patients (56.3, p < 0.001). The evaluation of clinical parameters revealed a predominance of anterior uveitis and late sequalae such as cataract and glaucoma among the RAU patients. In 15 of the patients a history of prior RuV infections could be confirmed. The study underlines the importance of long-term surveillance of RuV associated diseases that originate from infections before the introduction of RuV vaccination programs.

## Introduction

Uveitis is a process of intraocular inflammation that may involve different sections of the uveal tract. The resulting clinical manifestations, such as iridocyclitis and chorioretinitis, can affect either one or both eyes and may be ascribed to various causes [1–3]. Apart from systemic or localized immune mediated disorders, infections are key players in the etiology of uveitis and ultimately entail different or even opposing treatment strategies [4, 5]. Rubella virus (RuV) is a recognized causative agent of virus-associated intraocular inflammation, mainly in the form of anterior uveitis.

RuV is also under discussion for its potential persistence as an RNA virus in an unknown cellular reservoir within human patients. Reactivation of vaccine-derived RuV was reported in pediatric patients with primary immunodeficiency diseases (PID) caused by different genetic defects. In these patients, vaccine-derived RuV is associated with cutaneous and visceral granulomatous dermatitis [6, 7]. Recently, RuV-associated granulomatous dermatitis was reported in an adult immunodeficient patient, and in this case, for the first time, a wild-type RuV was identified as a trigger of the disease process [8]. Whereas RuV-associated granulomatous dermatitis is predominantly caused by reactivated vaccine-derived RuV, RuV-associated uveitis (RAU) cases are especially detected in younger patients with a history of a wild-type RuV infection [9]. The implementation of the RuV vaccination, including elimination programs in several WHO regions, requires constant monitoring of rubella cases. In this regard, surveillance of the persistent occurrence of rubella cases, and even local outbreaks in areas with insufficient or even no vaccination coverage, e.g. in China and Japan in 2019 [10, 11], also need to be considered.

RuV-associated Fuchs uveitis (FU) is also known as Fuchs uveitis syndrome, Fuchs heterochromic iridocyclitis, or Fuchs heterochromic uveitis. As first described by Quentin and Reiber, Fuchs uveitis is a recognized RuV-associated disease [12, 13]. However, diagnostic criteria differ somewhat between studies and not all RAU cases present as FU [14]. Other infections may cause a clinical picture resembling FU [15], as described, for example, in CMV infections within an Asian population [16] and on rare occasions for *Toxoplasma gondii* [17, 18] infections. A thorough analysis of the underlying etiology is hampered by very sparse amounts of material obtained by diagnostic puncture of the anterior chamber, often below 50 µl [15]. Laboratory diagnosis is thus mainly based on an increased RuV antibody index, indicative of an intraocular antibody synthesis subsequent to an infection or reactivation of RuV [12, 19].

Here we retrospectively describe a cohort of 159 patients that underwent a diagnostic analysis of their aqueous or vitreous humor in order to determine an infectious etiology of an underlying uveitis at a tertiary referral hospital in Germany between 2013 and 2019. Patients with a RuV-associated uveitis and non-RAU patients were compared with regard to clinical parameters. Additionally, the history of wild-type RuV infection and vaccination of the RAU patients is presented.

## Materials and methods

### Study population

159 patients who presented with different forms of uveitis between March 2013 and December 2019 were included in the study. Aqueous or vitreous humor samples were obtained via operation or puncture of the anterior chamber along with a paired serum sample (serum) at the discretion of the treating physician. Follow-up samples were regarded as one case. Clinical data were retrieved retrospectively from patient charts and included uveitis localization, glaucoma, and cataract. The classification of uveitis was reported according to the standardization of uveitis nomenclature (SUN) working group [20]. Symptoms or diseases other than uveitis were reported as “other”. Patients of the RAU cohort were randomly assigned a case number from 1 to 38. Patients of the non-RAU cohort were randomly assigned case numbers 39 to 159.

### Pathogen-specific IgG

IgG antibodies against herpes simplex virus (HSV 1/2 pool, HSV), varicella-zoster virus (VZV), cytomegalovirus (CMV), rubella virus (RuV), and *Toxoplasma gondii* (Toxoplasma) were determined with commercially available CSF ELISA-assays (Euroimmun, Lübeck, Germany) according to the manufacturer’s instructions on a Euroimmun Analyzer I. Aqueous and vitreous humor samples were pre-diluted (1:10) with assay specific sample buffer in order to compensate for low sample volume.

### Total IgG

The total IgG concentration was determined with a commercially available human IgG ELISA (Genway Biotech, San Diego, California, USA) according to the manufacturer’s instructions. The final dilutions used were 1:80.000 for serum and 1:10.000 for aqueous or vitreous humor. Assay specific diluent buffer was used for all dilution steps.

### Goldmann-Witmer coefficient

Pathogen specific IgG-antibodies (IgS) of aqueous or vitreous humor (H) and serum (B) were determined and set into quantitative relation to the total IgG (IgT). Coefficients greater than two were considered as pathologic. The Goldmann-Witmer coefficient (GWC) was calculated according to the formula:

1 $$ \text{G}\text{W}\text{C}=\frac{IgS\left(H\right)*IgT \left(B\right)}{IgS\left(B\right)*IgT\left(H\right)}$$

### Pathogen-specific IgM

In case of a pathologic GWC, IgM testing was performed for the respective pathogen. Commercially available assays for HSV, VZV, CMV, RuV (all medac, Wedel, Germany), or Toxoplasma (VIDAS, bioMeriéux Marcy-l’Étoile, France) were performed according to the manufacturer’s instructions.

### Pathogen-specific IgG avidity

In case of a pathologic GWC, IgG avidity testing was performed for the respective pathogen. Commercially available assays for HSV, VZV, RuV (all Enzygnost, Siemens Healthcare Diagnostic Products, Marburg, Germany), CMV (ARCHITECT, Abbott, Chicago, Illinois, USA), or Toxoplasma (VIDAS, bioMeriéux Marcy-l’Étoile, France) were performed according to the manufacturer’s instructions.

### Nucleic acid (NA) extraction and pathogen-specific NA testing

In case of a pathologic GWC, a published nucleic acid amplification test (NAAT) was performed for HSV [21], VZV [22], CMV [23], RuV [24], or Toxoplasma [25], respectively. Total NA was extracted from the remaining aqueous or vitreous humor using the NucliSENS easyMag instrument (bioMeriéux Marcy-l’Étoile, France) according to the manufacturer’s instructions (input volume 100 µl, output volume 25 µl). The NA samples were stored in aliquots at -80 °C until further use. In case of insufficient sample volume, the specimen was diluted to a total volume of 100 µl up to a dilution of 1:5. No NAAT was performed in case of lower sample volumes.

### Statistical analysis

Statistical analysis was performed using IBM SPSS Statistics for Windows, Version 27.0 (Armonk, NY: IBM Corp.). Continuous values were expressed as mean and categorical data as frequencies (percentages). Student’s t-test was performed to compare means. Chi-square or Fisher’s exact test were performed for categorical variables. All tests were two-tailed. A p-level of < 0.05 was considered significant. The total IgG-Boxplot was built with R [26] using the ggplot2 package [27].

## Results

### Specimen

In total 148 aqueous humor samples, 27 vitreous humor samples, and 173 paired sera samples were obtained from 159 individual patients. For four patients, samples of both eyes were taken and for 10 patients follow-up samples were collected.

### Total IgG

**Fig. 1:**
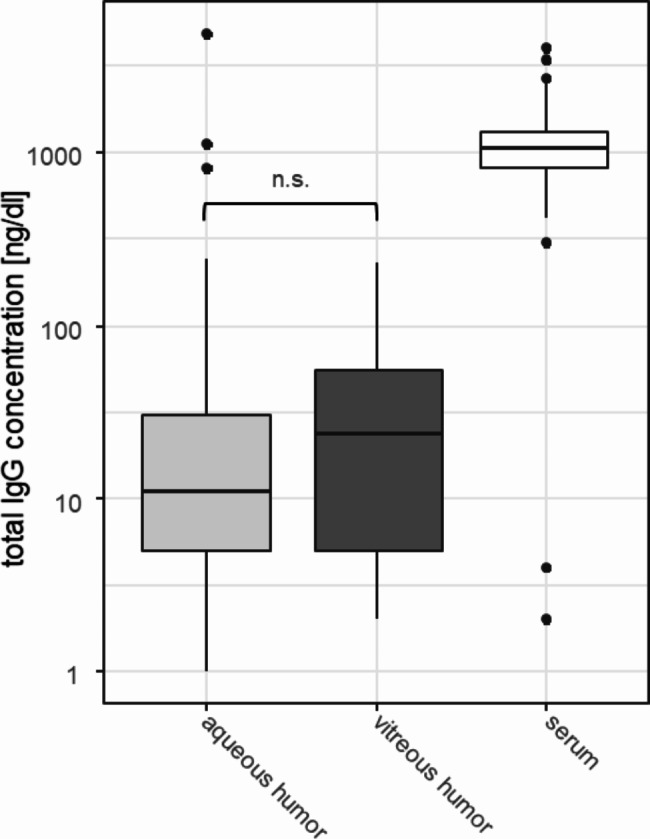
Total IgG concentration in aqueous humor (light grey, n = 148), vitreous humor (dark grey, n = 27), and serum samples (white, n = 173). The concentration of aqueous and vitreous humor samples was not statistically different (p = 0.554). (n.s.; not significant)

The mean total IgG concentrations and interquartile ranges were 24 [2–60] mg/dl for vitreous humor, 11 [5–31] mg/dl for aqueous humor, and 1064 [818–1328] mg/dl for serum samples (Fig. 1). The total IgG levels were not statistically different for vitreous or aqueous humor samples (p = 0.554).

### Serological analysis and pathogen-specific NAAT

The serological findings are presented in Table 1. The highest seroprevalence was found for VZV (98.7%) while it was lowest for CMV (61.6%). The prevalence of pathogen-specific IgG antibodies that could be detected in aqueous or vitreous humor was lower in comparison to the seroprevalence for all investigated pathogens.

A pathologic GWC was determined for 39 patients for RuV, 10 patients for VZV, eight patients for HSV, five patients for Toxoplasma, and three patients for CMV. In cases of a pathologic GWC, a reactive IgM could be identified only in five patients’ sera (all Rubella). In contrast, for all pathogens the specific IgG avidity was above 30%.

Due to the small sample volume, pathogen-specific NAAT was only performed in 66.2% (43/65) of the cases with a pathologic GWC (Table 1). At least one positive NAAT result could be obtained for HSV, VZV, CMV, and Toxoplasma. All 30 NAAT for the presence of RuV genomes were negative.

**Table 1:** Serological and NAAT analysis

			Rubella	VZV	HSV	CMV	Toxoplasma
seroprevalence		[%(n/total)]	94.7 (151/159)	98.7 (157/159)	82.4 (131/159)	61.6 (98/159)	69.2 (110/159)
intraocular antibody prevalence		[% (n/total)]	62.9 (100/159)	62.3 (99/159)	64.8 (103/159)	42.1 (67/159)	39.6 (63/159)
Goldmann-Witmer coefficient ≥ 2		[% (n/total)]	24.5 (39/159)	6.3 (10/159)	5 (8/159)	1.9 (3/159)	3.1 (5/159)
monocular		[% (n/total)]	92.3 (36/39)	90 (9/10)	100 (8/8)	66.6 (2/3)	80 (4/5)
binocular		[% (n/total)]	7.7 (339)	10 (1/10)	0 (0/8)	33.3 (1/3)	20 (1/5)
Serum IgM reactivity		[% (n/total)]	12.8 (5/39)	0 (0/10)	0 (0/8)	0 (3/3)	0 (5/5)
Serum IgG avidity below 30%		[% (n/total)]	0 (0/39)	0 (0/10)	0 (0/8)	0 (3/3)	0 (5/5)
positive NAAT		[% (n/total)]	0 (0/30)	40 (2/5)	66.6 (2/3)	33.3 (1/3)	50 (1/2)

Analyzed categories are displayed on the column to the left and are given as frequencies (%). (n/total) indicates the respective cases of the total amount of available data. Due to low sample volume, a nucleic acid amplification test (NAAT) could be performed only for a subset of cases

Five patients showing a pathological GWC for more than one pathogen were identified. Patient 72 showed a pathologic GWC for RuV (2.3) and VZV (4.9) and was diagnosed with multiple sclerosis. Consequently, the patient was not classified as having RAU. Four patients had pathologic GWCs for HSV and VZV. For patient 121, the initial GWCs were 4.5 and 9.9, respectively, with a concomitant positive NAAT result for VZV (82.000 copies/ml). In a follow-up sample, a pathologic GWC for VZV (18.5) in addition to a decreased viral load of VZV (5 copies/ml) were detected. For patients 40, 47, and 98 the respective GWCs for HSV and VZV were 28.9 and 8.7, 4.9 and 6, and 15.5 and 58, respectively.

### Study population

The mean age of the study population was 53.8 with a female proportion of 57.2%. Of the 159 examined patients, 38 were assigned to the RAUgroup according to the GWC analysis. 121 patients were assigned to the non-RAU group (Table 2). Patients of the RAU group were younger than the non-RAU patients with a mean age of 45.9 and 56.3, respectively (p < 0.001). Patients with RAU predominantly showed an anterior uveitis in comparison to the non-RAU group, while isolated posterior uveitis and panuveitis were rare (p = 0.002 and p = 0.004, respectively). Cataract (p < 0.001) and glaucoma (p = 0.016) were more frequent in the RAU group. In the RAU group, the majority of infections involved only one eye (94.7%) compared to 66.9% in the non-RAU group (p < 0.001).

**Table 2:** Clinical characteristics of the study population

		RAU	Non-RAU	Total	*p*-Value
***study population***					
cases	[% (n/total)]	23.9 (38/159)	76.1 (121/159)	100 (159/159)	n.a.
female	[% (n/total)]	36.8 (14/38)	44.6 (54/121)	42.8 (68/159)	0.397
male	[% (n/total)]	63.2 (24/38)	55.4 (67/121)	57.2 (91/159)	
age [years]	[mean ± SD]	45.9 ± 10.1	56.3 ± 21.5	53.8 ± 19.9	**< 0.001**
***Goldmann-Witmer coefficient ≥ 2***					
Rubella	[% (n/total)]	100 (38/38)	0.8 (1/121)	24.5 (39/159)	**< 0.001**
VZV	[% (n/total)]	0 (0/38)	8.26 (10/121)	6.3 (10/159)	0.067
HSV	[% (n/total)]	0 (0/38)	6.6 (8/121)	5 (8/159)	0.129
CMV	[% (n/total)]	0 (0/38)	2.5 (3/121)	1.9 (3/159)	0.327
Toxoplasma	[% (n/total)]	0 (0/38)	4.1 (5/121)	3.1 (5/159)	0.256
***clinical presentation***					
uveitis					
anterior	[% (n/total)]	100 (38/38)	28.1 (34/121)	45.3 (72/159)	**< 0.001**
intermediate	[% (n/total)]	18.4 (7/38)	11.6 (14/121)	13.2 (21/159)	0.277
posterior	[% (n/total)]	2.6 (1/38)	25.6 (31/121)	20.1 (32/159)	**0.002**
panuveits	[% (n/total)]	0 (0/38)	19 (23/121)	14.5 (23/159)	**0.004**
cataract	[% (n/total)]	63.2 (24/38)	12.4 (15/121)	24.5 (39/159)	**< 0.001**
glaucoma	[% (n/total)]	36.8 (14/38)	18.2 (22/121)	22.6 (36/159)	**0.016**
other	[% (n/total)]	7.9 (3/38)	36.4 (44/121)	29.6 (47/159)	**< 0.001**
***localization***					
monocular	[% (n/total)]	94.7 (36/38)	66.9 (81/121)	73.6 (117/159)	**< 0.001**
binocular	[% (n/total)]	5.3 (2/38)	33.1 (40/121)	26.4 (42/159)	

Analyzed categories are displayed on the column to the left and are either given as frequencies (%) or as means and standard deviations (mean ± SD). (n/total) indicates the respective cases for the total amount of available data. The brackets indicate parameters that were analyzed in the same contingency table. Statistically significant p-values (p < 0.05) are presented in bold. (n.a., not applicable)

### RAU cohort

The number of patients that were diagnosed with RAU were two in 2013 (5.3%), four in 2014 (10.5%), five in 2015 (13.2%), nine in 2016 (23.7%), seven in 2017 (18.4%), eight in 2018 (21%), and two in 2019 (5.3%). The clinical parameters in addition to the history of vaccination and wild-type RuV infection of the RAU patients were extracted from patients’ charts (Table 3). For 48.4% (15/31) of the RAU patients, an infection with RuV was reported. In 29% (9/31) of the cases, there was a history of rubella vaccination. In seven cases both a vaccination as well as a wild-type RuV infection was documented. Wild-type RuV infections were documented in early childhood for all patients with a history of wild-type RuV infection (n = 15) and preceded the rubella vaccinations in patients where both infection and vaccination were documented (n = 7). For seven patients no information regarding RuV infection or vaccination was available.

**Table 3:**
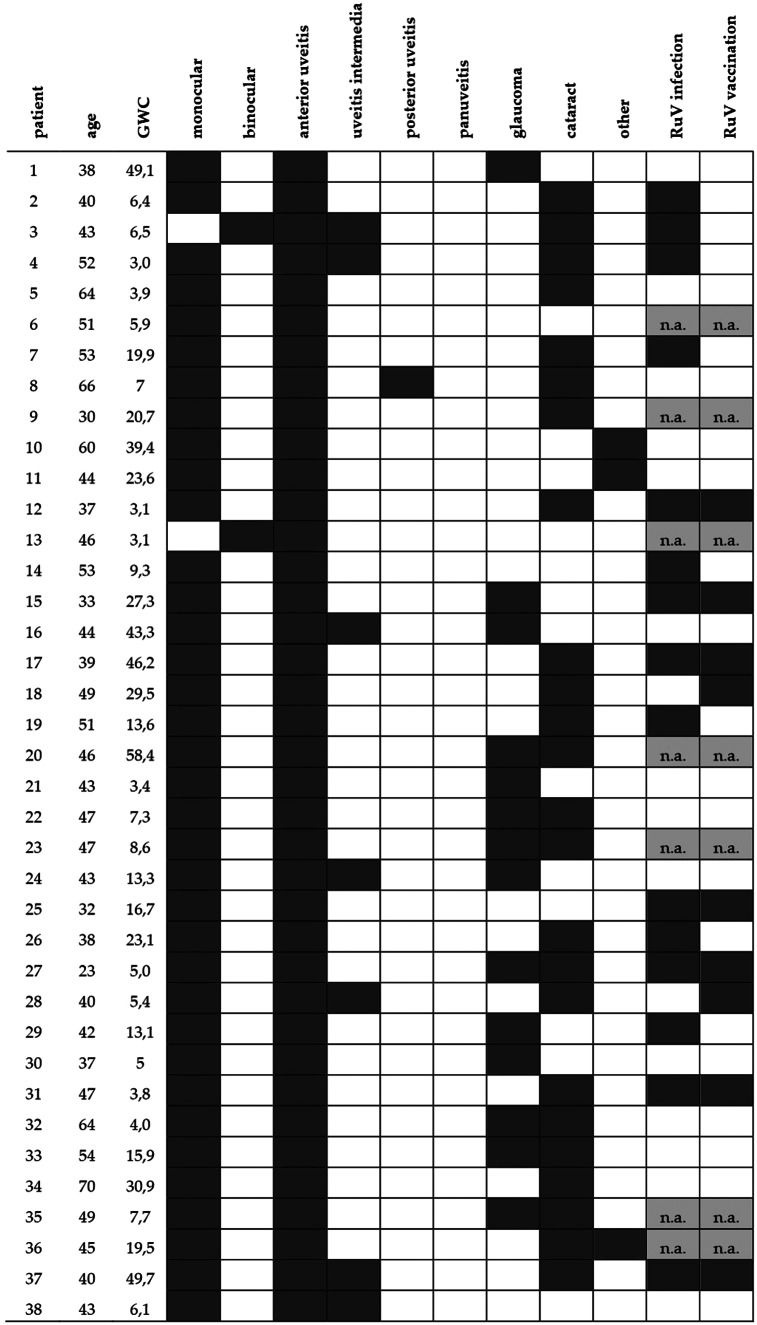
Clinical characteristics of the RAU patients

Each row represents an individual patient and each column the indicated parameter. If a parameter was present, the respective box is depicted in dark grey, otherwise it is shown in white. If no data was available, the box is marked in light grey (n.a., not available)

## Discussion

Diagnosis of Rubella-virus associated uveitis is challenging due to low sample volume, low viral load in the eye, and limitations in detection of RuV genomes [15]. Quantitative detection of RuV was described only in a minority of cases, including the report by Quentin and Reiber on the detection of RuV genome in 18% of Fuchs heterochromic cyclitis samples [12] as well as the report by Groen-Hakan showing RuV PCR-positive results in only 20% of the patients [14]. Thus, the most important parameter of RuV-associated uveitis diagnosis is the presence of RuV antibody synthesis in the eye [12]. Interestingly, a recent study by Gonzales indicates that the unbiased approach of metagenomic deep sequencing is superior to reverse transcriptase PCR targeting the E1 protein [28]. Notably, all samples tested positive for genes of the nonstructural open reading frame, whereas genes encoding the structural proteins were detected in only half of the analyzed samples. It could be hypothesized that only parts of the viral genome, the so-called defective interfering or DI RNAs, persist after RuV infection. RuV DI RNAs generated in cell culture lack the entire or most of the structural protein open reading frame [29], which could contribute to the low rate of viral genome detection by reverse transcriptase PCR targeting a specific viral structural protein.

Suitable and effective treatment options for RAU and FU are still lacking. Corticosteroid treatment is still in use, but as already suggested by Quentin & Reiber, it is not only ineffective, but is also associated with an increased risk for the development of cataract and glaucoma [12]. Moreover, there is still some time interval between symptom onset and diagnosis, which was also noted for the present cohort showing late sequelae of chronic inflammation such as glaucoma and cataract. Thus, with our study we emphasize RuV as a cause of uveitis and stress the importance of its laboratory diagnosis especially in settings of an unspecific eye inflammation. Two cases with an initial clinical suspicion of FU were eventually identified as Posner–Schlossman syndrome due to normal GWCs for RuV, thus representing a relevant aspect for differential diagnosis.

In comparison to non-infectious uveitis and cataract, RAU has a well-defined cytokine profile, which is followed by infiltration of T cells and monocytes/macrophages [30]. However, a comprehensive evaluation of a large patient cohort with virus-associated anterior uveitis in association with CMV, HSV, VZV, and RuV revealed no significant differences in the addressed 27 immune mediators for RuV in comparison to the other viruses [15].

The age distribution of the patient cohort with 45.9 ± 10.1 years was in the range of other published studies: 44 years for a German [15] and 43.9 ± 14.3 years for a US patient cohort [31]. However, a Chinese cohort reported a lower median age of 30.3 ± 11.1 at the onset of the symptoms and of 31.5 ± 10.8 at the diagnosis of Fuchs uveitis [32]. This may suggest an influence of the genetic background on the onset of FU, which occurs between the third until the end of the fourth/beginning of the fifth decade.

So far there is only one report in the literature on a successful isolation of RuV from the aqueous humor of a patient with Fuchs uveitis [33]. The authors discussed the source of RuV in the aqueous humor as acute infection, reinfection or even reactivation of latent virus. There are still open questions on the issue of possible reactivation mechanisms of the latent RNA virus RuV. In this regard, it is noteworthy that an early work on the susceptibility of human embryonic organ cultures showed growth of RuV in fetal lenses but not in adult lenses [34]. This study suggests that the adult lens is not accessible during postnatal RuV infection. Thus, reactivation of RuV and the onset of RuV-associated uveitis could potentially occur in different cell populations. Moreover, although human retinal pigment epithelia are susceptible to RuV infection, infectious virions were detected by transmission electron microscopy after co-cultivation with Vero cells as an activation step [35]. This could at least partially explain the difficulties in isolation of RuV from uveitis cases.

The effective vaccination program and the vaccination-associated decrease in the number of FU patients in the USA [31] highlight RuV vaccination as a preventive treatment of RuV-associated uveitis. However, the association of its reactivation under immunosuppression may at least raise some concern for the future. Vaccination against RuV was started in some countries, including Germany, after the first RuV vaccine was licensed in 1969 [10]. Particularly in Germany, RuV vaccination was introduced for girls in 1973 and became a regular vaccination for all children in 1980. A second dose was recommended in 1991 [36]. While in China the vaccination program was started in 2010, other countries are still lacking a RuV vaccination program [10]. We suggest continuing surveillance of RuV in association with FU and viral anterior uveitis until clinical data is available on the possible reactivation of a live attenuated vaccine in the presence of an aging immune system. It needs to be discussed that as vaccinated people age, the immune system is less effective [37] and thus vaccine strains of RuV could be reactivated. Just recently a case of RuV FU was reported for a vaccinated patient in association with common variable immunodeficiency [38].

There are several limitations of this study: Due to low specimen volumes of aqueous and vitreous humor, a pathogen-specific NAAT could not be performed for all patients with a pathologic GWC. Additionally, false negative NAAT test results cannot be ruled out as a nucleic acid extraction was done with prediluted samples in a subset of the patients. Due to the long latency to the development of RuV-associated uveitis and a lack of documentation of childhood diseases, the percentage of wild-type RuV infections is likely to be underestimated in the RAU cohort.

## Conclusions

Besides its nature as a highly teratogenic virus after prenatal transmission during pregnancy and as a causative agent of a mild childhood disease, wild-type infection may lead to RuV-associated uveitis as a distinctive and late-onset clinical characteristic of postnatal RuV infection, which warrants continuous future awareness in the treatment of uveitis.

## Data Availability

The data that support the findings of this study are not openly available due to reasons of participant confidentiality and are available from the corresponding author upon reasonable request. Data are located in controlled access data storage at Leipzig University.
